# Tailoring carbon nanotubes optical properties through chirality-wise silicon ring resonators

**DOI:** 10.1038/s41598-018-29300-1

**Published:** 2018-07-26

**Authors:** Elena Durán-Valdeiglesias, Weiwei Zhang, Carlos Alonso-Ramos, Samuel Serna, Xavier Le Roux, Delphine Maris-Morini, Niccolò Caselli, Francesco Biccari, Massimo Gurioli, Arianna Filoramo, Eric Cassan, Laurent Vivien

**Affiliations:** 1 Centre for Nanoscience and Nanotechnology, CNRS, Univ. Paris-Sud, Université Paris-Saclay, C2N - Orsay, 91405 Orsay cedex, France; 2 Department of Physics, University of Florence. European Laboratory for Non-linear Spectroscopy, 50019 Sesto Fiorentino (FI), Italy; 3 CEA Saclay, IRAMIS, NIMBE (UMR 3685), LICSEN, Bât. 125, F-91191 Gif-sur-Yvette, France; 4 Present Address: Optoelectronics Research Centre, University of Southampton, Southampton, SO17 1BJ UK

## Abstract

Semiconducting single walled carbon nanotubes (s-SWNT) have an immense potential for the development of active optoelectronic functionalities in ultra-compact hybrid photonic circuits. Specifically, s-SWNT have been identified as a very promising solution to implement light sources in the silicon photonics platform. Still, two major challenges remain to fully exploit the potential of this hybrid technology: the limited interaction between s-SWNTs and Si waveguides and the low quantum efficiency of s-SWNTs emission. Silicon micro-ring resonators have the potential capability to overcome these limitations, by providing enhanced light s-SWNT interaction through resonant light recirculation. Here, we demonstrate that Si ring resonators provide SWNT chirality-wise photoluminescence resonance enhancement, releasing a new degree of freedom to tailor s-SWNT optical properties. Specifically, we show that judicious design of the micro-ring geometry allows selectively promoting the emission enhancement of either (8,6) or (8,7) SWNT chiralities present in a high-purity polymer-sorted s-SWNT solution. In addition, we present an analysis of nanometric-sized silicon-on-insulator waveguides that predicts stronger light s-SWNT interaction for transverse-magnetic (TM) modes than for conventionally used transverse-electric (TE) modes.

## Introduction

Driven by their outstanding electrical properties, semiconducting single walled carbon nanotubes (s-SWNTs) are being widely investigated for the realization of ultra-compact field effect transistors (FET), reaching the nanoscale^1,2^, with very high on-off current ratios^3^. Nevertheless, s-SWNTs also exhibit very interesting optical properties. For instance, s-SWNT are direct band gap semiconductors providing absorption, photo- and electro-luminescence at room temperatures from the visible to the mid-infrared wavelengths, which makes them a suitable material for the realization of on-chip light sources and detectors^4–7^. Indeed, just by properly choosing the applied voltage, the same s-SWNT-based device can be operated as a FET, light emitting diode (LED) or photodetector^8^. In addition, carbon nanotubes can have exceptional thermal conductivity that has already been exploited to demonstrate electrically driven emitters with direct modulation speeds in the GHz range^9^. Furthermore, s-SWNTs present intrinsically fast nonlinear Stark and Kerr effects^10,11^ that could enable the realization of low-power-consumption high performance electro-optical modulators. In addition, SWNTs have been widely used for the implementation of high-performance saturable absorbers in mode-locked lasers^12–14^. This features pose s-SWNTs as a very interesting nano-material for the realization of compact optoelectronic devices. Among the photonic platforms to exploit the outstanding properties of s-SWNT, silicon photonics has several advantages: its mature large-volume fabrication processes, ultra-compact optical waveguides and potential for integration with microelectronics. Hence, the hybrid s-SWNT integration on silicon photonic platform could open a new route towards the widespread of compact on-chip opto-electronic transceivers.

The band gap of s-SWNT is determined by their chirality. In fact, s-SWNT can have emission and absorption wavelengths all along the datacom (around 1300 nm wavelength)^15–17^ and telecom (around 1500 nm wavelength)^8,18,19^ ranges. Thus, proper s-SWNT chirality selection is a key feature for the realization of performing photonic devices. In this context, polymer-assisted selection techniques are a very interesting approach yielding high-purity s-SWNT solutions (with negligible traces of metallic carbon nanotubes)^20^ that are compatible with wafer-scale deposition processes^21^. Nonetheless, these solutions typically contain various s-SWNT chiralities. For instance, using Poly-9,9-di-n-octyl-fluorenyl-2,7-diyl (PFO) it is possible to select s-SWNTs with chiralities of (8,6), emitting around 1200 nm wavelength, and (8,7), emitting around 1300 nm wavelength. However, single s-SWNT chirality selection remains a challenge^22–24^.

Resonance enhancement of s-SWNT emission has been reported in micro-ring resonators^25,26^ and photonics crystal cavities^17,27^, that exploit tight light confinement and resonant light recirculation to maximize device-light-SWNT interaction. In this work, we demonstrate that integration with Si micro-ring resonators also provides SWNT chirality-dependent interaction enhancement, allowing further tunability of the optical properties of hybrid Si-s-SWNT photonic devices. We experimentally demonstrated that a proper design of the micro-resonator geometry allows promoting the resonance enhancement of either (8,6) or (8,7) s-SWNT chiralities present in PFO-sorted s-SWNT solution. These results open a new route towards single-chirality selection in hybrid Si-SWNT devices, even if the SWNT solution contains various chiralities.

**Figure 1:**
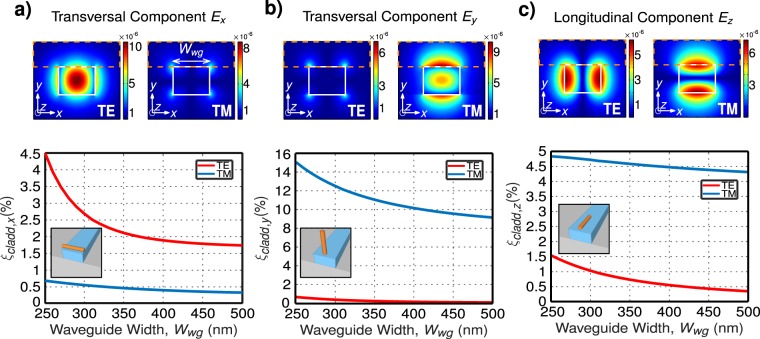
Electric field distribution for waveguide width of *W*_*wg*_ = 350 nm, normalized to the total mode power, and figure of merit *ξ*_*cladd*,*p*_ as a function of waveguide width for (**a**) transversal *E*_*x*_, (**b**) transversal *E*_*y*_ and (**c**) longitudinal *E*_*z*_ components of both fundamental TE and TM modes at wavelength of 1300 nm. Insets schematically show preferred SWNTs orientation for maximized light-SWNTs interaction for each component of the electric field.

Interaction between s-SWNT and Si photonic waveguides is governed by the s-SWNT geometry and the waveguide mode field distribution. The very large aspect ratio of s-SWNTs, with nanometer-scale diameters and micrometer-scale lengths, makes them behave like dipoles. This means that the maximum emission (and absorption) occurs for an electromagnetic field aligned to the long s-SWNT axis^28,29^. Therefore, Si photonic waveguides aiming to efficiently interact with s-SWNTs should support modes with a large evanescent field parallel to the s-SWNTs. Conventional Si waveguides support quasi-transverse electric (TE) modes, with electric field (transversal to the light propagation) oriented parallel to the chip surface, and quasi-transverse magnetic (TM) modes, with transversal electric field perpendicular to the chip surface. Most of the s-SWNT deposition methods (drop casting, spin coating, dielectrophoresis) result in networks of s-SWNTs arranged along the chip surface. Thus, previous works on the integration of s-SWNTs and Si photonic structures focused on the use of TE modes. This way, s-SWNTs have been efficiently coupled to the TE modes of conventional strip waveguides^30^, slot^26^ and strip^31^ micro-ring resonators or 1-D^27^ and 2-D^17^ photonic crystal cavities. However, TE modes concentrate most of the evanescent field in the vertical waveguide walls (see Fig. 1a), having a strong interaction with the sidewall roughness arising from the etching process, thus resulting in comparatively high propagation loss. Conversely, TM modes concentrate most of the evanescent field in the horizontal walls (see Fig. 1b), thereby allowing lower propagation loss^32^. In addition, for the same waveguide dimensions, e.g. 220 nm thick and 350 nm wide waveguide, TM modes are typically more delocalized, having a larger overlap with the surrounding medium. The problem is that optimizing the interaction between s-SWNTs and the dominant component of the electric field in TM modes (perpendicular to the chip surface) would require placing the s-SWNTs vertically on the waveguide top surface (see inset in Fig. 1b). This ideal scenario, although possible, is technologically challenging and compromises the feasibility of the approach. Here, we propose a new route to circumvent this limitation by exploiting the hybrid nature of the optical modes in SOI waveguides with sub-wavelength scale core dimensions. The proposed approach is to exploit the longitudinal electric field component in TM modes to interact with SWNTs aligned parallel to the chip surface, thereby obviating the need for technologically challenging vertical SWNT deposition. Owing to the vast index difference between Si and SiO_2_ and the ultra-tight confinement in 220-nm-thick SOI nanowires, (quasi-)TM modes can have a strong electric field component longitudinal to the light propagation. This longitudinal mode field component, parallel to the chip surface, can be advantageously exploited to interact with s-SWNTs. Indeed, we show that TM modes can provide a longitudinal electric field on top of the Si waveguides (where the s-SWNTs are placed), equal or even larger than the transverse electric field in TE modes. Here, we exploit the large index contrast of the SOI platform and waveguide geometry engineering to demonstrate that, opposite to the common knowledge, TM optical modes can efficiently interact with drop-casted s-SWNTs arranged along the chip surface. We also developed a selective deposition technique that provides simple and tight control of the regions where s-SWNTs interact with the Si waveguides, minimizing detrimental light absorption from non-excited SWNTs. We theoretically and experimentally studied the s-SWNTs interaction with SOI micro-ring resonators, implemented with strip waveguides optimized to maximize the longitudinal TM component and optimized to yield resonant-enhancement at specific wavelength ranges corresponding to either (8,6) or (8,7) s-SWNT chiralities.

## Results

First, we optimize the waveguide cross section to maximize the longitudinal TM component, *E*_*z*_, on top of the waveguide. To do so, we follow the conventional approach in active hybrid Si photonic devices and calculate the dielectric energy confinement in the active region, SWNT in this case^33^. We define the figure of merit *ξ*_*cladd*,*p*_ which accounts for the percentage of the dielectric energy confinement on top of the Si waveguide (slashed region in Fig. 1). We calculate *ξ*_*cladd*,*p*_ for each electric field component (*E*_*p*_ with *p* = *x*, *y*, *z* for the *E*_*x*_, *E*_*y*_ and *E*_*z*_ components respectively as

1 $${\xi }_{cladd,p}=\frac{{\int }_{cladd}\varepsilon ({\rm{x}},{\rm{y}}){|{E}_{p}({\rm{x}},{\rm{y}})|}^{2}{\rm{dxdy}}}{{\int }_{total}\varepsilon ({\rm{x}},{\rm{y}})[{|{E}_{x}({\rm{x}},{\rm{y}})|}^{2}+{|{E}_{y}({\rm{x}},{\rm{y}})|}^{2}+{|{E}_{z}({\rm{x}},{\rm{y}})|}^{2}]{\rm{dxdy}}},$$

where *ε* is medium permittivity. As conventionally done when modeling these polymer-sorted SWNTs^17,25,26^, we considered a homogeneous top cladding with a refractive index of 1.6 (similar to that of polyfluorene^34^), which emulates the scenario of drop casting deposition. We fixed the Si waveguide thickness to 220 nm, and varied the waveguide width from 250 nm to 500 nm (*W*_*wg*_ in Fig. 1). Figure 1 shows the *ξ*_*cladd*,*p*_, calculated with an eigenmode solver tool^35^, as a function of the waveguide width for a wavelength of 1300 nm. The transversal *E*_*y*_ component from the TM mode has the largest figure of merit, ${\xi }_{cladd,y}\sim \mathrm{12 \% }$. However, optimal interaction with the *E*_*y*_ field would require vertically aligned SWNTs (see inset of Fig. 1b), which makes this solution technologically challenging. Conversely, for both, transversal *E*_*x*_ and longitudinal *E*_*z*_ components, the preferred SWNTs orientations are parallel to the chip surface (see inset of Fig. 1a and c), which makes them very interesting for scenarios relying on planar deposition techniques (drop casting, spin coating, etc). According to our calculations, the figure of merit for the *E*_*z*_ component in the TM mode can be larger than that of *E*_*x*_ component in the TE mode, which is conventionally optimized for hybrid SWNT integration. We then chose a waveguide width of *W*_*wg*_ = 350 nm, that presents a figure of merit of ${\xi }_{cladd,z}\ge \mathrm{4.5 \% }$ (with ${\xi }_{cladd,x}\sim \mathrm{0.5 \% }$ and ${\xi }_{cladd,y}\sim \mathrm{12 \% }$), close to the maximum for TM mode with a total dielectric energy confinement in the cladding of ∼20%. Moreover, this waveguide width provides single-mode operation and good optical confinement that keep low losses and sharp bending. Electric field distributions for all components of TE and TM modes for the selected waveguide are shown in the top panels of Fig. 1.

**Figure 2:**
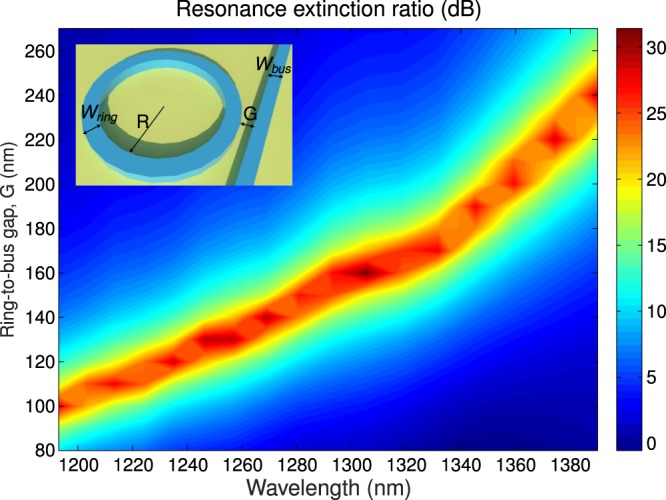
Simulated resonant extinction ratio for TM modes, defined as the ratio between on-resonance and off-resonance transmission, considering *G* between 80 nm and 260 nm. Inset: Schematic view of micro-ring resonator with bus waveguide.

Then we shaped up the TM response of micro-ring resonators implemented with the optimized waveguide. The goal was to yield a strong light matter interactions in a specific bandwidth, matching the emission range of a given s-SWNT chirality. By adjusting the geometry of the micro-ring, it was then possible to tune the optimal micro-resonator wavelength, releasing an extra degree of freedom to selectively promote light s-SWNT interaction for only (8,6) or (8,7) s-SWNT present in our solution, with emission around 1200 nm and 1300 nm, respectively. As schematically depicted in the inset of Fig. 2, we used an all-pass ring-resonator configuration where the ring resonator waveguide, with waveguide width of *W*_*ring*_, was evanescently coupled to an access strip waveguide (width of *W*_*bus*_), separated from the ring by a gap distance *G*.

The resonant behavior of a coupled micro-ring resonator is strongly dependent on the coupling between the ring and the bus waveguide. Light recirculation inside the ring (hence energy storage, and electric field enhancement) is maximal when coupling between the ring and the bus equals the loss inside the ring (critical coupling condition)^36^. Aiming to yield selective resonance enhancement of the SWNT emission, we decided to implement an asymmetric coupling scheme. To do this, we set a bus waveguide width of *W*_*bus*_ = 270 nm and ring waveguide width of *W*_*ring*_ = 350 nm (providing strong *ξ*_*cladd*,*z*_). As the bus and ring waveguides have different widths, modes propagating through them have different phase propagation constants, precluding perfect phase matching. This results in a lower power coupling between the waveguides and a shorter beat-length (distance for maximum power transfer between the waveguides). For the short couplers used here, the coupling strength is directly proportional to the overlap between the modes of the bus and ring waveguides. The mode profile is more deconfined for longer wavelengths, resulting in larger overlaps, therefore stronger coupling. On the other hand, it can be shown that the bandwidth of the coupler is related to the beat-length dispersion^37^. Thus, this asymmetric configuration yields a strong chromatic dispersion of the bus-to-ring coupling ratio, i.e., for a given bus-to-ring gap (*G*), critical coupling condition is achieved for a comparatively narrow bandwidth. Hence, we can choose the wavelength range where resonant light recirculation (thus light-SWNT interaction) is maximized just by properly choosing the gap *G* between the ring and the bus waveguide. We chose a ring radius of *R* = 5 *μ*m, that yields a free spectral range (wavelength separation between consecutive resonances) of ∼12 nm, easily discernible with standard spectrometer. Figure 2 shows the resonance extinction ratio for TM modes, calculated with the finite difference time domain (FDTD) tool^35^. It can be seen that critical coupling condition (corresponding to deep resonances with extinction ratio greater than 30 dB) was achieved for narrow wavelength ranges of _∼_10 nm, with optimum range moving towards shorter wavelengths for decreasing gap widths.

We fabricated different ring resonators varying the bus-to-ring gap (*G*) between 80 nm and 270 nm. Tapered waveguides are used to inject/extract light through the chip facet, with a coupling loss of ∼10 dB. This insertion loss may be reduced, e.g. by implementing high-efficiency fiber-chip grating couplers^38^. Propagation loss of the Si waveguides is in the 3–5 dB/cm, range, which is compatible with envisioned datacom and telecom applications^39^. As described in Methods, we defined interaction windows in the coupling region between the bus waveguide and ring resonator and deposited a high-purity s-SWNT solution.

**Figure 3:**
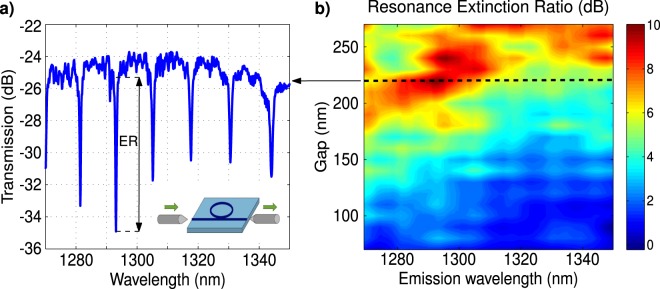
Measured linear spectral response for TM mode of Si micro-ring resonators with bus waveguide width of *W*_*bus*_ = 270 nm, ring waveguide width of *W*_*ring*_ = 350 nm, ring radius of *R* = 5 *μ*m for (**a**) fixed bus-to-ring gap of *G* = 220 nm, and (**b**) bus-to-ring gap varying between 80 nm and 270 nm. Dashed line indicates position of spectrum for gap *G* = 220 nm, shown in (**a**).

The linear response of the devices was experimentally characterized using a tunable laser and automatic data acquisition system, CT400 from Yenista (see Methods and the inset in Fig. 3a). Input light polarization was set by a polarization controller. Light was injected and extracted from the chip using tapered Si waveguides and lensed fibers (∼10 dB loss per facet). Note that because of the limited range of our tunable source (between 1260 nm and 1350 nm) we were not able to inspect the region around 1200 nm wavelength, corresponding to the emission of (8,6) s-SWNT. Figure 3b shows the measured resonance extinction ratio in our micro-rings with drop-casted s-SWNT, as a function of the wavelength for different bus-to-ring gaps. The optimum wavelength range shifts to shorter wavelengths for decreasing gaps. Results in Fig. 3 show that the critical coupling condition is met for a narrow wavelength range, with optimum wavelength that increases with the gap, in good agreement with our calculations. The ring resonators with s-SWNTs yield quality factors for TM modes of $Q\sim 4000$ and extinction ratio of ∼10 dB at around 1300 nm wavelength for a bus-to-ring gap between 200 nm and 250 nm. On the other hand, the best quality factor for TE polarization is around $Q\sim 4000$, with a gap of 100 nm. The difference with calculated values shown in Fig. 2, can be attributed to scattering losses due to sidewall roughness and absorption in the s-SWNT, not considered in the simulations. Similar ring resonators without s-SWNT typically yield quality factor, *Q*, up to 10000. The small interaction window, of 1.5*μ*m radious used for the deposition of SWNTs did not induce excess loss to the ring transmission. However, the perturbation produced by the presence of the s-SWNTs (mainly due to absroption loss) is enough to reduce the quality factor of the resonances.

**Figure 4:**
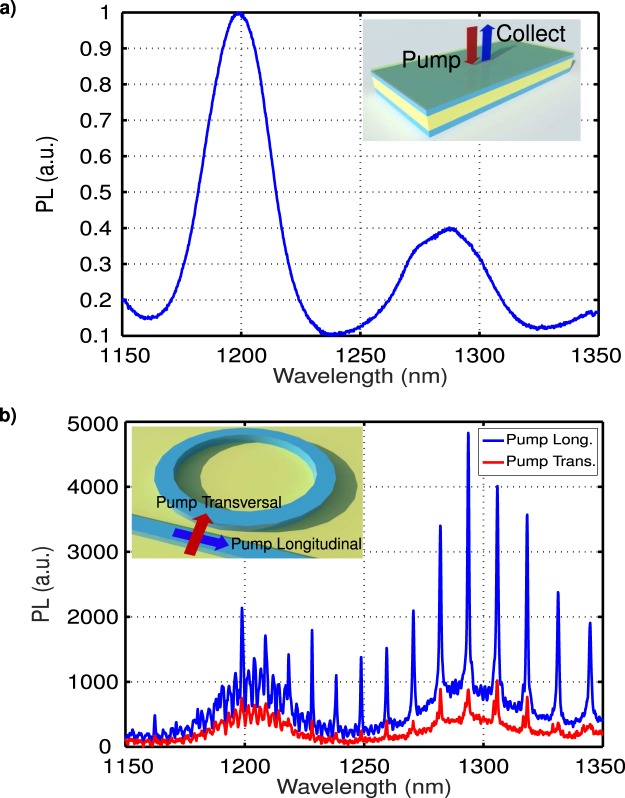
(**a**) Normalized photoluminescence signal of SWNTs solution drop casted on unpatterned SOI sample. Excitation wavelength of 735 nm with vertical excitation/collection. Inset: Schematic view unpatterned SOI sample with a scheme of excitation of SWNTs and collection of the PL from surface. (**b**) Photoluminescence spectrum for a ring resonator of radius *R* = 5 *μ*m and bus-to-ring gap *G* = 250 nm. The excitation is performed from the chip surface with a wavelength of 735 nm and the generated photoluminescence signal coupled to bus waveguide is collected from the chip facet. Collected photoluminescence signal when pump laser is transversal and longitudinal with respect to the propagation axis are represented in red and blue lines respectively. Inset: Schematic view of ring resonator coupled to a bus waveguide with both polarization schemes, transversal (aligned with the *E*_*x*_ component) and longitudinal (parallel to *E*_*z*_ component).

Then the resonant enhancement of the s-SWNTs photoluminescence in the ring resonators was characterized. For comparison, Fig. 4a shows the normalized photoluminescence of the high purity s-SWNTs solution deposited onto an unpatterned SOI sample under pumping excitation at wavelength of 735 nm. The s-SWNTs emission presented two wideband emission regions around 1200 nm and 1300 nm wavelengths corresponding to the two main s-SWNT chiralities available in the sample, (8,6) and (8,7), respectively. The characterization of the photoluminescence response of our Si micro-ring resonators was performed by pumping the s-SWNTs layer from the surface, focusing the light coming from a Ti:Sa laser with an objective. We used an objective microscope with a magnification of 50X and NA of 0.55 that generated an excitation beam with a radius of ∼1.5 *μ*m. We collected the generated photoluminesce at the facet of the chip using a polarization-maintaining lensed fiber and polarization splitter to discern contribution from TE and TM modes. TE and TM modes have different effective index and group index. This means that ring resonances, thus emission enhancement, occur at different wavelengths with a different free spectral range (distance between consecutive resonances). Therefore, if there was some TE signal passing through the polarization splitter, its contribution would be shifted compared with respect to the TM one. Note that illuminating the SWNTs with the Ti:Sa laser changes their absorption properties, offsetting the optimal coupling gap, compared with the linear measurements shown in Fig. 3. Here, we compared the photoluminescence spectrum of a micro-ring resonator with gap of *G* = 250 nm, in the optimal photoluminescence enhancement region for 1300 nm wavelength, when the polarization of the excitation beam was aligned with the transversal component (perpendicular to the waveguide) or longitudinal *E*_*z*_ component (aligned along the waveguide). As shown in Fig. 4b, tuning the pump beam polarization from transversal (*E*_*x*_) to longitudinal (*E*_*z*_) components it was possible to favor the excitation of the TM modes in the ring. These results are in good agreement with near-field scanning experiments that exploited a similar effect to image TM modes in Si micro-ring resonators^40^. In the following experiments we kept the pump beam aligned with the longitudinal *E*_*z*_ component.

**Figure 5:**
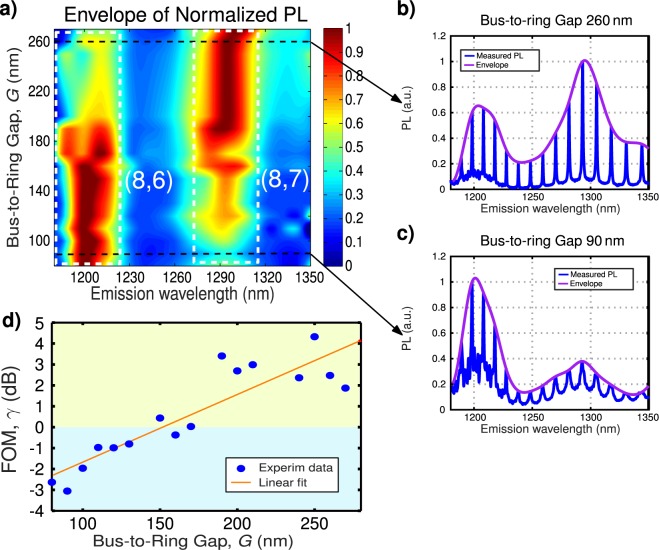
(**a**) Normalized photoluminescence spectrum of SWNTs deposited on Si micro-ring resonators with bus waveguide width of *W*_*bus*_ = 270 nm, ring waveguide width of *W*_*ring*_ = 350 nm, ring radius of *R* = 5 *μ*m, and bus-to-ring gap, *G* varying between 80 nm and 270 nm. SWNTs are excited at 735 nm wavelength from the chip surface using a microscope objective and the generated PL is collected from the chip facet with polarization maintaining lensed fiber. Detail of collected PL for (**b**) G = 260 nm, and (**c**) G = 90 nm. (**d**) Figure of merit estimated from measurements.

In Fig. 5a we plot the upper envelope of the photoluminescence signal collected at the output bus waveguide for different gaps between the ring and the bus waveguide. Each of the rings will have slightly different resonant wavelength (mainly arising from small fabrication imperfections), by representing upper envelope we remove this spurious effect, yielding smoother plots that make the gap effect clearer. In Fig. 5b and c we show two examples of measured spectrum and calculated upper envelope. From Fig. 5a, it is apparent that, as expected from the micro-ring design, smaller gaps yield stronger photoluminescence signal for (8,6) s-SWNTs, while larger gaps promote emission from (8,7) chirality. Note that the photoluminescence signal around 1250 nm wavelength is weak, compared to 1200 nm and 1290 nm wavelength where (8,6) and (8,7) chiralities emit. Thus, it is not possible to clearly see the smooth resonant wavelength shift predicted by simulations in Fig. 2.

To compare the photoluminescence resonant enhancement for (8,6) and (8,7) chiralities, we have defined the resonance enhancement factor, *α*, as the ratio between on-resonance (*I*_*ON*_) and off-resonance (*I*_*OFF*_) intensities, as

2 $$\alpha =\frac{{I}_{ON}}{{I}_{OFF}}.$$

We computed this factor for wavelengths around 1200 nm, *α*(1200), and 1290 nm, *α*(1290). Then we defined the figure of merit, *γ*, as the ratio between the resonance enhancement factors for this two wavelengths:

3 $$\gamma =10\,\mathrm{log}(\frac{\alpha (1290)}{\alpha (1200)}).$$

In Fig. 5d we display the figure of merit *γ*, estimated from measurements, as a function of the bus-to-ring gap. Smaller gaps favor resonance enhancement of s-SWNTs with (8,6) chirality ($\gamma  < 0$), with emission around 1200 nm wavelength, while larger gaps promote the resonant enhancement of s-SWNTs with (8,7) chirality ($\gamma  > 0$), with emission around 1300 nm wavelength. Furthermore, we can observe in Fig. 5d how the maximum enhancement, smoothly shifts towards longer wavelengths, as the gap increases, in good agreement with calculations shown in Fig. 2.

## Discussion

The outstanding electrical and optical properties of s-SWNT make them a very promising solution for photonics and more especially for the silicon photonics. Despite the great development of polymer-based s-SWNT processing techniques, yielding quasi-impurity-free solutions, single SWNT chirality selection remains an issue. In this work, we experimentally demonstrated that by integrating the s-SWNT onto Si micro-ring resonators, it is possible to realize SWNT chirality-wise resonant enhancement of the emission, allowing an added SWNT chirality selection mechanism. We experimentally show, for the first time, that by combining a novel selective deposition process (based on localized interaction windows and drop casting) and engineered Si waveguides for the ring resonator and bus waveguide, we selectively promote the resonant enhancement of either (8,6) or (8,7) s-SWNT chiralities present in our solution. Unlike previously reported hybrid Si-SWNT devices^17,27,30,31^, the ring resonators presented here exploit the strong longitudinal electric field component of the TM mode to interact with the SWNT. By providing selective chirality selection and efficient interaction with TM modes, these results unlock two new degrees of freedom to implement s-SWNT-based devices for the silicon photonics platform. This technique could be further improved in the future, e.g. by implementing slot ring resonators that may result in nearly a 60% resonant enhancement increase^26^.

## Methods

The s-SWNTs were prepared using a polymer-sorting technique that provides high purity solutions containing s-SWNTs emitting around 1200 nm wavelength, chirality of (8,6), and 1300 nm wavelength, chirality of (8,7)^41^. We started the process with a commercial s-SWNT powder (HiPCO from Unydim), mixed with the polymer Poly-9,9-di-n-octyl-fluorenyl-2,7-diyl (PFO, Sigma-Aldrich) and toluene. The mixture, with the ratio of s-SWNT (5 mg): PFO (20 mg): toluene (30 ml), was homogenized by sonication and the supernatant solution was collected after 1 hour of ultracentrifugation at 150000 g. Finally, we drop-casted the purified s-SWNTs solution onto the Si chip and annealed 15 minutes at 180 °C.

The ring resonators were fabricated in silicon-on-insulator (SOI) wafers with 220 nm thick silicon film and 2 *μ*m thick buried oxide layer. The photonic structures were defined by electron beam lithography followed by inductively coupled plasma etching. A hydrogen silses-quioxane (HSQ) cladding, with refractive index of ∼1.45 and thickness of ∼800 nm, was deposited by spin coating and an additional lithography step was performed to form small circular aperture in the coupling regions between the micro-ring resonators and the bus waveguides. The HSQ cladding isolated the waveguide from the deposited s-SWNTs everywhere except in the interaction windows, which have a diameter of 3 *μ*m, matching the size of the Ti:Sa excitation spot for pumping. This scheme ensured that all s-SWNTs interacting with the Si micro-resonators were under the excitation illumination, minimizing extra losses arising from unwanted absorption from non-excited s-SWNTs.

The linear response of the micro-ring resonators was characterized by injecting the light from a tunable Yenista laser source (1260 nm–1350 nm wavelength) and collecting the transmitted signal with polarization maintaining lensed fibers through input and output waveguides at the chip facets. The responses of the devices were collected using an automatic data acquisition system, CT400 from Yenista. Polarization of the light injected into the chip was controlled with a polarization rotator and polarizer at the input. To characterize the resonant enhancement of SWNTs photoluminescence, SWNTs were excited from the surface of the chip with a continuous wave Titane Sapphire (Ti:Sa) laser and the generated photoluminescence coupled to the waveguide was collected at the chip facet through the output bus waveguide using a polarization maintaining lensed fiber. The Ti:Sa excitation beam passed through a polarization beam splitter, to control the polarization state, through a low pass wavelength filter, to remove higher order harmonics of the laser, and was finally focused onto the chip surface with a microscope objective (50x, NA = 0.55, excitation beam diameter of ∼1.5 *μ*m). The pump power, measured before the objective, was 1.5 mW and the wavelength was chosen to match the S22 excitonic transition of SWNTs (around 735 nm wavelength). The collected spectrum was analyzed with a 320 mm long spectrometer with a 950 lines/mm grating, coupled to a nitrogen-cooled InGaAs array with 512 pixels. All experiments were realized at room temperature and air condition.
